# Identifying non-essential uses to phase out substances of very high concern under REACH

**DOI:** 10.3389/ftox.2024.1488336

**Published:** 2024-11-01

**Authors:** Flora Borchert, Romain Figuière, Ian T. Cousins, Christina Rudén, Marlene Ågerstrand

**Affiliations:** Department of Environmental Science, Stockholm University, Stockholm, Sweden

**Keywords:** essential use, application for authorisation, REACH, risk management, risk assessment, SVHC, chemical strategy for sustainability, green deal

## Abstract

The essential use concept aims to better protect consumers, vulnerable groups, and the environment from the most harmful chemicals by phasing out uses considered non-essential for society. Given the lack of empirical research evaluating this novel approach for chemical management in real-world settings, the aims of the present analysis were to 1) investigate if the information provided in applications for authorisation under REACH allowed for the identification of non-essential uses of substances of very high concern (SVHCs), and 2) identify data gaps, challenges and potential needs for revising the assessment criteria to effectively implement the essential use concept in the REACH authorisation. In total, 100 uses covering 11 SVHCs were analysed. 4-(1,1,3,3-tetramethylbutyl) phenol (OPnEO) and chromium trioxide were among the most frequently used substances, covering 42% and 35% of the analysed uses. Using the current essential use criteria, 55% of all analysed uses were categorised as essential, while 10% were categorised as non-essential. Potentially, authorisations would not have been granted for the identified non-essential uses under REACH if the concept had been implemented at the time. However, for 35% of the uses it was not possible to assess their essentiality and these uses were therefore categorised as “complex.” These challenges were due to the multiple purposes of the technical function, lack of detailed information on the spectrum of end-uses, and difficulties in interpreting the essential use criteria. Consequently, for a successful implementation of the essential use concept, we recommend the European Commission to develop guidance for applicants and refine the essential use criteria to ensure a transparent and resource-efficient authorisation procedure under REACH.

## 1 Introduction

Inspired by the Montreal Protocol from 1987, which regulates the use of ozone-depleting substances, scientists recently suggested that the so-called “essential use concept” could be applied more broadly to other harmful chemicals. Following this, the European Commission announced in 2020 its ambition to prioritise phasing out *the most harmful substances* unless their use is *essential for society* (European Commission, 2020a; United Nations Environment Programme, 2022a; United Nations Environment Programme, 2022b; Cousins et al., 2019). *The most harmful chemicals* are defined in the Chemicals Strategy for Sustainability Towards a Toxic-Free Environment (further the Chemical Strategy for Sustainability) as chemicals classified as carcinogenic, mutagenic, toxic to reproduction, persistent, bioaccumulative, affecting the endocrine, immune, neurological or respiratory systems, and/or toxic to a specific organ (European Commission, 2020a). Aiming at simplifying the phase-out of these chemicals, the essential use concept is deemed to contribute to improved transparency and predictability of the regulatory process (European Commission, 2020a; WSP E&IS GmbH, 2023). In the Chemical Strategy for Sustainability, the European Commission stated that the essential use concept would be applied across all relevant regulations, including the REACH Regulation (EC 1907/2006). An ongoing discussion addresses the question of how this concept, as a novel approach to risk management, should be implemented. This discussion involves consultations on how to define “essential use,” estimating the impacts on regulatory processes, and which information should drive decision-making (WSP E&IS GmbH, 2023; Garnett and Van Calster, 2021; Figuière et al., 2023). A 2023 report by WSP E&IS GmbH (further referred to as WSP), on behalf of the European Commission, aimed to further define the essential use concept and associated criteria to assess the feasibility of applying the concept “horizontally” across chemicals legislation in the EU (WSP E&IS GmbH, 2023). One year later, the European Commission employed these suggestions as the basis in their communication on *Guiding criteria and principles for the essential use concept in EU legislation dealing with chemicals* (European Commission, 2024).

Under REACH, the use of substances of very high concern (SVHCs) can be controlled under the authorisation procedure. It aims to “*ensure a good functioning of the internal market while assuring that the risks from substances of very high concern are properly controlled,”* and to progressively replace them with *“suitable alternative substances or technologies where these are economically and technically viable”* (European Commission, 2006) (REACH Article 55). SVHCs are substances classified as carcinogenic, mutagenic or toxic to reproduction in categories 1A or 1B (CMR), persistent, bioaccumulative and toxic (PBT), very persistent and very bioaccumulative (vPvB), or endocrine disrupting chemicals (EDCs) and chemicals of equivalent concern (REACH Article 57). Thus, they are covered by the definition of *the most harmful chemicals* [see above, (European Commission, 2020a)]. If an SVHC is listed in Annex XIV of REACH, i.e., the authorisation list, a company that would like to continue using the SVHC has to apply for authorisation before a defined sunset date. This can be done either by proving that risks are well under control (adequate control route), or by showing that the socio-economic benefits of using the SVHC outweigh the risks (socio-economic route). In an obligatory assessment of alternatives and a substitution plan, the applicant must show that there are currently no suitable alternatives available that pose a lower risk overall and that are economically and technically feasible (ECHA, 2011a; ECHA, 2021; European Commission, 2020b).

At the time of writing, the essential use concept stipulates that the use of *the most harmful substances* would only be allowed if two criteria are met: 1) the use is “*necessary for health/safety or critical for the functioning of society,*” and 2) “*there are no alternatives that are acceptable from the standpoint of environment and health*” (European Commission, 2020a). If both criteria are met, the use would be deemed essential for society and, if the concept were to be implemented in the REACH authorisation procedure, authorisation would be granted for a limited time (WSP E&IS GmbH, 2023). In comparison to the REACH authorisation, and also in contrast to the Montreal Protocol, the essential use concept, as currently stipulated in the Chemical Strategy for Sustainability, does not explicitly include economic aspects in the evaluation of essentiality.

Figuière and colleagues (2023) performed an analysis of the REACH authorisation procedure and suggested that the essentiality of a specific use could be assessed using the information already required and provided in today’s authorisation process, meaning there would be no need for major changes to the existing legislation and procedures (Figuière et al., 2023; ECHA, 2011a; ECHA, 2021). Since the European Commission emphasized prioritising the phase-out of the most harmful chemicals, it needs to be clarified whether the essential use concept would be capable of identifying non-essential uses. Therefore, the present analysis aimed to 1) investigate if the information provided by applicants in applications for authorisation allowed for the identification of non-essential uses, and 2) identify data gaps, challenges and potential needs for revising the assessment criteria to effectively implement the essential use concept in the REACH authorisation.

## 2 Method

### 2.1 Scope, assumptions and definitions of the essential use concept in the present analysis

In their report, WSP refined the stepwise “horizontal” approach for the decision-making on the (non-)essentiality of a use as suggested by the European Commission (WSP E&IS GmbH, 2023). During the work with this paper, the European Commission published their official criteria (European Commission, 2024). These criteria differ only in minor ways that do not affect the current analysis, e.g., rephrasing definitions without changing their meaning by shortening text, adding examples and/or using synonyms. It was therefore decided to continue using the WSP criteria, the principles of their approach, their described scope and assumptions, and to apply them in the context of the REACH authorisation process (see also Supplementary Table S2). A full description thereof can be found in the supplementary information section of the paper. The main aspects of the scope of, and assumptions made within, the essential use concept for the present analysis are summarised below:

- The evaluation focused on the SVHCs subject to REACH authorisation (Annex XIV), for which applications for authorisation have been submitted to the European Chemicals Agency (ECHA), as they are a sub-set of the most harmful chemicals as defined in the Chemical Strategy for Sustainability (European Commission, 2020a).
- The use of an SVHC is determined by its technical function to provide certain properties during an industrial process, or to products or articles resulting from that process, however, that alone is not sufficient to determine the essentiality. The technical function of an SVHC in the context of the end-use of the application for authorisation and its potential essentiality to health/safety and/or the functioning of society needs to be considered and is addressed in the present analysis. Following the WSP report, *necessary*, *critical* and *essential* were interpreted as synonyms in the present analysis (see Supplementary Table S2 in the Supplementary Material).
- Although only the use of an SVHC is addressed in an application for authorisation and not the SVHC or an article as such, the context of the end-use (articles, mixtures, products, processes and/or services) should be considered in order to be able to apply the concept (WSP E&IS GmbH, 2023). This can be for example, a final product or article resulting from the use of an SVHC during the industrial process which is employed in the context of medical treatment but does not contain the SVHC itself.
- As one of the conditions to get an authorisation is that there are no suitable alternatives available to the applicant (Figuière et al., 2023), it has been assumed that there are no ready-to-use alternatives available to the applicants for the SVHC to replace the required technical function. Hence, no evaluation of the analysis of alternatives part of the application was performed.

A set of essentiality criteria regarding health/safety and the functioning of society were used to analyse the selected applications for authorisation (Table 1). They were adopted from the WSP report and are based on an analysis of existing legislation and literature, as well as consultations with relevant stakeholders (WSP E&IS GmbH, 2023). In their report, WSP describes *necessary* and *critical* as the dependency of something to be achieved. In the context of an essentiality assessment, our interpretation is that the use of an SVHC could be considered essential if its technical function in the context of the end-use fulfils the criteria for essentiality (WSP E&IS GmbH, 2023). This includes considering the impact of not using a certain substance, i.e., the impact of losing the technical function it provides, on health/safety or the functioning of society. According to the WSP report, other social and economic factors are not supposed to be considered when evaluating if the criteria are fulfilled (e.g., loss of jobs, impacts on a company’s economy) ((WSP E&IS GmbH, 2023), Supplementary Table S2). In their communication on *Guiding criteria and principles for the essential use concept in EU legislation dealing with chemicals*, it is stated that the EU Commission “*does not intend to change existing references to a technical and/or economic feasibility assessment if it proposes to introduce the essential use concept in any such legislative area. The Commission will weigh up the appropriateness of such references to the legislative context when considering the introduction of the concept of essential use in any other areas*” (European Commission, 2024). They emphasize that an evaluation of economic feasibility is deemed to be part of an alternative assessment. However, economic aspects are not an explicit part of the EU Commission’s essential use criteria as such and it is yet unclear how, and by who, social and economic aspects will be assessed, weighed against each other and integrated into an essentiality assessment. Therefore, even though we acknowledge that it would be crucial step in the decision process, an analysis of the social and economic impacts as part of an application for authorization were excluded from the current analysis.

**TABLE 1:** Overview of the essential use criteria and their definitions, according to the WSP report (WSP E&IS GmbH, 2023). The footnotes are according to the table provided in the WSP report.

Essential use criteria	Definition
Necessity for health/safety	
Prevention, monitoring or treatment of severe health issues	The WHO definition for health: A state of complete physical, mental and social wellbeing and not merely the absence of disease or infirmity^a^. Any acute or chronic illness and/or health condition that carries a high risk of mortality negatively impacts the quality of life and daily function, and/or is burdensome in symptoms, treatments, or caregiver stress^b^, including severe mental illness that prevents from engaging in functional and occupational activities. Risk factors linked to severe health issues, e.g., household air pollution, unsafe water, lead exposure and others listed by the Institute for Health Metrics and Evaluation/Risk Factors Collaborators under the Global Burden of Disease study^c^. Severe noncommunicable diseases^d^, e.g., cardiovascular diseases, cancer, respiratory diseases and diabetes, as well as severe but less prevalent health issues, are included, too. May include uses in medical devices, pharmaceuticals, healthcare, or other health-related uses, also beyond the healthcare sector, e.g., hygiene and cleaning or physical exercise.
Sustainment of basic conditions for human life and health	Basic needs for human life and health: food and water security, adequate housing. Environmental health as a prerequisite for good human health.
Management and prevention of health crises and emergencies	Outbreaks of diseases, emergencies covered by ambulance services, etc.
Personal safety	Ensuring personal safety, e.g., the proper functioning of seatbelts, personal protective equipment (sports, workplace), bulletproof vests, life jackets, fire alarms, etc. Ensuring fire resistance in products supposed to be heated, ensuring lubrication in vehicle brakes, etc.
Public safety	Safety of public infrastructure, e.g., road safety, and public building safety, ensure the effective functioning of emergency services through, e.g., military, police, anti-terrorism, cyber security, and fire safety services.
Addressing a danger to animal health which cannot be contained by other means	Safeguarding animal health and welfare according to EU standards, prevention and control of parasites/diseases (including zoonoses), pest control, prevention/minimisation of animal suffering.
Criticality for the functioning of society	
Provision of resources or services which are critical for society	Installation and maintenance of critical infrastructure which are “organisational and physical structures and facilities of such vital importance to a nation’s society and economy that their failure or degradation would result in sustained supply shortages, significant disruption of public safety and security, or other dramatic consequences”^e^, e.g., energy and transport, as well as waste treatment, water treatment, communication infrastructure, healthcare infrastructure, obtaining/storing critical raw materials^f^.
Management of societal risks and impacts from natural and man-made crises and emergencies	E.g., repairing/preventing damage to infrastructure from natural or man-made disasters
Protection of cultural heritage	Monuments, groups of buildings and sites that are of Outstanding Universal Value^g^ from the point of view of history, art/aesthetics, science, ethnology and/or anthropology, and provide cultural diversity, social capital, collective belonging and other service.
Running of traditional and religious practices	Uses in products applied in traditional and religious practices, as in the Minamata Convention on Mercury^h^.
Protecting and restoring the natural environment	Protection and restoration of the natural environment, ecosystem services in particular, reduction of greenhouse gas emissions, biodiversity loss, analysis/monitoring/remediation of pollutants.

^a^ World Health Organisation, WHO. Constitution of the World Health Organization. Accessed 2022–11–22 at: https://www.who.int/about/governance/constitution

^b^ Radbruch et al., 2020. Redefining Palliative Care—A, New Consensus-Based Definition. Journal of Pain and Symptom Management. 60, 754–764.

^c^ The Lancet. Global Burden of Disease 2019 risk factor summaries. Accessed 2022–06–26 at: https://www.thelancet.com/gbd/summaries

^d^ World Health Organization, WHO (2022). Noncommunicable diseases: Key facts. Accessed 2022–06–26 at: https://www.who.int/news-room/fact-sheets/detail/noncommunicable-diseases

^e^ according to the Federal Office of Civil Protection and Disaster Assistance (BKK) of Germany, National Strategy for Critical Infrastructure Protection (KRITIS strategy). Accessed 2023–10–30 at: https://www.bsi.bund.de/EN/Themen/KRITIS-und-regulierte-Unternehmen/Kritische-Infrastrukturen/Allgemeine-Infos-zu-KRITIS/allgemeine-infos-zu-kritis_node.html

^f^ European Commission. Critical raw materials. Accessed 2022–06–28 at: https://single-market-economy.ec.europa.eu/sectors/raw-materials/areas-specific-interest/critical-raw-materials_en

^g^ United Nations Educational, Scientific and Cultural Organization, UNESCO (2021). Operational guidelines for the.

implementation of the World Heritage Convention. Accessed 2023–06–28 at: https://whc.unesco.org/en/guidelines/

^h^ Accessed 2023–06–29 https://mercuryconvention.org/sites/default/files/2021-06/Minamata-Convention-booklet-Sep2019-EN.pdf

### 2.2 Selection of applications for authorisation for analysis under REACH

**FIGURE 1:**
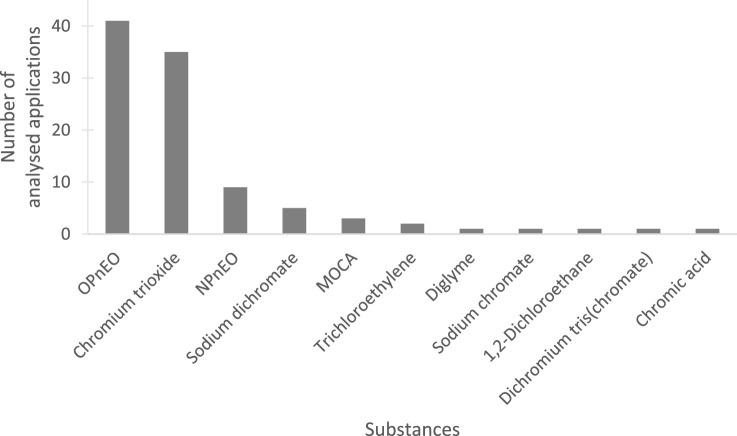
Overview of the 100 analysed applications for authorisation and uses of 11 SVHCs. OPnEO = 4-(1,1,3,3-tetramethylbutyl) phenol, ethoxylated, NPnEO = 4-nonylphenol, branched and linear, ethoxylated, MOCA = 2,2′-dichloro-4,4′-methylenedianiline/4,4′-methylenebis [2-chloroaniline].

ECHA’s database on *Adopted opinions and previous consultations on applications for authorisation* (ECHA, 2024) was accessed on 2 November 2022. This database lists all applications for authorisation that were submitted for substances listed on Annex XIV, i.e., substances subject to authorisation. Information from 454 applications for authorisation that were submitted for the continued use of 30 SVHCs, were extracted into an Excel spreadsheet. From that pool, applications which contained a substitution plan were selected for analysis (n = 173). In 2020 the rules changed, and since then a substitution plan must be part of an application (European Commission, 2020b; ECHA, 2022). Hence, it has been assumed that the applications that include a substitution plan are the ones with the most recent and comprehensive set of information. Applications for which ECHA’s Committee for Risk Assessment (RAC) and Committee for Socio-Economic Analysis (SEAC) were still developing their opinion at the time of the present analysis were not evaluated (n = 33). Furthermore, two applications were void, or the applicants ceased their use prematurely, and one was withdrawn by the applicant. Finally, 37 applications were merged (e.g., 0143–02 and –03 were merged with 0143–01) as the applicants described similar uses of an SVHC with the same technical functions but in different applications. A final pool of 100 applications for authorisation covering 11 SVHCs was analysed (Figure 1).

### 2.3 Analysis of the selected applications for authorisation

For the identification of non-essential uses, information was extracted from the following parts of the applications for authorisation (ECHA, 2011a; ECHA, 2021):

• Use-related descriptions and general information by the applicant displayed on ECHA’s webpage of the respective application for authorisation,
• The non-confidential version of the socio-economic analysis (SEA), or joint SEA and analysis of alternatives (AoA), if available,
• Opinions on the application for authorisation by RAC and SEAC.

The detailed criteria for data collection are provided in the Supplementary Table S2 “Method.” All information was collected in an Excel spreadsheet (Supplementary Table S1).

#### 2.3.1 Necessity of the use for health/safety and its criticality for the functioning of society

In the context of the stepwise approach suggested by WSP (Section 2.1, and Supplementary Material), we analysed if the technical function provided by the SVHC in the context of the end-use, as described in the applications for authorisation, fulfilled the criteria for essential use as defined by WSP (Table 1). Based on the collated information from the applications, particularly the socio-economic analysis part, the uses were categorized as follows:

- *Essential*: A use was categorised as “essential” if the purpose of the technical function of an SVHC for an end-use fulfilled the essential use criteria, i.e., it was covered by the technical functions defined as essential in the WSP report (Table 1). For example, if the only purpose of the technical function of an SVHC used as a corrosion inhibitor was to provide drinking water safety for all end-uses (such as faucets in households), then the use would be considered to fulfil the criterion *Prevention of […] severe health issues: Unsafe water* and *water safety.* In such a case, it was assumed that drinking water safety would be impacted by a loss of the technical function provided by that SVHC (see Section 2.2 on the definition of *essential*, *critical* and *necessary*), and, hence, the use of the substance would be considered as essential.
- *Non-essential*: In a “non-essential” use case, the purpose of the technical function of an SVHC in an end-use did not fulfil any of the essential use criteria.
- *Complex*: A use was categorised as “complex” when a decision on a category could not be made due to e.g., the (lack of) information on the purpose of the technical function(s) provided by the SVHC and/or a failure to interpret the criteria, i.e., to decide if the technical function fulfilled the WSP criteria (Table 1).

A description of these assessments is reported as free text in the Supplementary Table S1.

## 3 Results

### 3.1 Overall description of the analysed applications for authorisation and SVHCs

The majority of SVHCs were carcinogenic, mutagenic and/or toxic to reproduction (10/11), while two of them, OPnEO (4-(1,1,3,3-tetramethylbutyl) phenol, ethoxylated) and NPnEO (4-nonylphenol, branched and linear, ethoxylated), were classified as endocrine disruptors. Most applications were submitted for OPnEO (42% of 100 applications) and chromium trioxide (35%) (Figure 1). Usually, the applicants were downstream users (90%), covering only their use of the SVHC (91%, 82/90). So-called upstream applications, in which the manufacturer, formulator or importer of the SVHC applied for the downstream use of the substance, accounted for 9% of applications. More than one applicant was applying for authorisation in 16% of applications. Most applicants, either individually or as a group, were situated in Germany (31%), France (18%), Italy (14%) and Spain (10%). Applicants from non-EU countries were involved in 9% of all applications. The annual use volumes indicated in the applications ranged from less than 1 kg (application 0161–01 OPnEO) to 3,000 kg (application 0032–03 chromium trioxide). The total sum of use volumes was approximately 9,766 metric tonnes per year. For almost all applications the socio-economic route was chosen to justify continuing the use of an SVHC (98%). The review period for which an authorisation is granted was requested by the applicants and agreed on by RAC and SEAC in 74.3% of applications and accounted for 10.7 years on average. More information can be found in the Supplementary Table S1 “Data extraction.”

### 3.2 Identified non-essential uses of SVHCs

**FIGURE 2:**
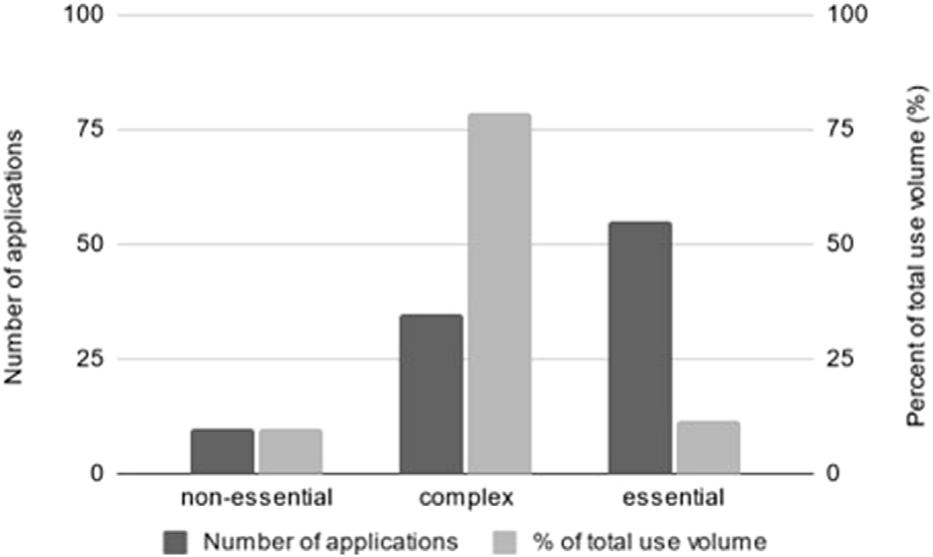
The dark grey bars present an overview of the numbers of applications for authorisation under REACH in which the uses applied for were categorised as non-essential (10/100), complex (35/100) or essential (55/100) according to the essential use criteria suggested in the WSP report (2023). The light grey bars indicate the use volume of each category of the total use volume in percent (%).

In 10 applications, i.e. 10 % of those analysed, uses were categorised as non-essential (Figure 2), meaning that authorisation would potentially not have been granted if our interpretation of the essential use criteria had been applied. This proportion equals nearly 950 tonnes of non-essential uses of SVHCs, accounting for 9.7% of the total use volume of the analysed applications. No article category was stated in 8 of the 10 uses deemed as non-essential. The substances affected were chromium trioxide (8/10) and sodium dichromate (2/10).

In these uses, chromium trioxide is used in the automotive sector to pre-treat (etch) and chrome-plate plastic articles used as interior and exterior design elements in (premium) cars, such as, e.g., grilles, spoilers, internal consoles, and brand labels. The technical function of chromium trioxide is twofold. First, as an etching agent to oxidize and roughen the plastic articles’ surface. Second, it is applied as a plating agent to react with the plastic articles’ surface and to provide a “*high-class surface*” (e.g., application 2018–01) of aesthetic value that is resistant to corrosion, abrasion, sunlight and chemicals. Hence, one SVHC provides different functions. It should be noted that chromium trioxide includes hexavalent chromium, which is responsible for the carcinogenic properties of the substance. However, once the chromium trioxide has reacted with the plastic surface, only elementary chromium is present. This means that the actual SVHC is not present in the final article for consumer use. Consequently, some applicants indicated that the substance would not lead to inclusion into or onto an article, hence, no article category was stated (e.g., applications 0071–01 and 0244–02). However, workers are exposed to the SVHC during industrial use. Since the technical function of chromium trioxide in these uses does not fulfil the criteria for health/safety and/or the functioning of society, these uses were categorised as non-essential.

Sodium dichromate is used as a mordant/chelating agent for the dyeing of textiles black and other dark colours, mainly wool (applications 0105–01 and 0222–01). The substance forms a complex with the dye which then attaches to the textile that is used in the fashion industry sector to produce clothes for consumer use. Through the chemical reaction, no carcinogenic hexavalent chromium is deemed to be detectable in the final product (<0.5 mg/kg wool). The technical function is needed for aesthetic reasons to “*ensure an excellent wet fastness and a depth of tone and intensity that cannot be reached with other dye molecules*” other than chrome-based dyes to satisfy customer demands (application 0105–01). Similar to chromium trioxide, the technical function of sodium dichromate in its uses did not fulfil the criteria for health/safety and/or the functioning of society, which is why these uses were categorised as non-essential given the assumptions made in Section 2.1.

### 3.3 Identified essential uses

Uses identified as essential accounted for 55% of the analysed applications (Figure 2), with nonylphenols OPnEO (39/55) and NPnEO (9/55) accounting for 87.3% of these. Other uses covered chromium trioxide (3/55) and sodium dichromate (2/55), as well as dichromium tris(chromate), sodium chromate, and trichloroethylene (1/55, each). Essential uses mostly provided a technical function for a medical end-use and summed up to approximately 1,132 tonnes per year, accounting for 10% of the total use volume. Representative examples of essential uses are described in the following.

Regarding the technical function, OPnEO and NPnEO were both used either as surfactants (sometimes referred to as detergents) or emulsifiers (sometimes referred to as dispersants). Despite describing the same critical properties, the technical function had to deliver, some applicants used different terms to name the technical function (e.g., “*Dispersing agent, processing aid, surfactant, stabilising agent*” in application 0186–03, “*lysing agent*” in application 0193–01, “*Cell disruption*” in application 0205–01). When used as a surfactant, the two nonylphenols lyse cell membranes, stabilise target proteins and inactivate unwanted viruses. These properties are needed in buffers or reagents applied in *in vitro* diagnostics (IVD) for the monitoring and detection of various diseases and the production of active pharmaceutical ingredients for treatment (e.g., cancer, HIV, arthritis, rare immune system or bleeding disorders, applications 0162–01, 0176–01, 0180–01, 0190–01, 0198–01), as well as for the production of vaccines (e.g., against influenza, application 0156–01) and other medicinal products (e.g., human blood for transfusions, application 0155–01). The technical function provided by OPnEO and NPnEO ensures the health safety of the end products and that they comply with medical and pharmaceutical regulations, fulfilling criterion *Prevention, monitoring or treatment of severe health issues: Uses in medical devices, pharmaceuticals, healthcare, or other health-related uses*.

The use of chromium trioxide as a corrosion inhibitor in the functional chrome plating of engine valves and valve actuation (“*lash adjusters*”) is another example of an essential use (AfA 0228–01). The engine valves are parts of passenger cars and heavy-duty trucks, while the valve actuations are only parts of passenger cars. Through the chrome plating, the applicant states that the valves can move smoothly, are wear-resistant, can endure high temperatures during engine running and ensure a higher endurance and proper functioning of the engine overall. The technical function of chromium trioxide in this use fulfilled the criteria for *personal safety* as well as *public safety* (road and transportation safety) (Table 1).

In applications 0236–01 and 0136–01, sodium dichromate and sodium chromate are used as anticorrosion agents in gas absorption heat pumps. Their technical function provides resistance to corrosion for sealed circuits within the pumps, effectiveness at high operating temperatures, high functionality under high pressure and a long-lasting service of the gas absorption heat pumps. This technology is a novel heating system that is deemed to produce approximately 40% less carbon dioxide emissions than common heating systems and to be crucial in achieving climate goals set by European governments to decarbonise the heating sector. In particular, residual buildings are deemed as the major contributors to emissions caused by heating. Hence, the technical function of sodium dichromate and sodium chromate fulfilled the criterion *Protecting and restoring the natural environment: Reduction of greenhouse gas emissions by means of reducing carbon dioxide emissions*.

### 3.4 Identified complex uses of SVHCs

Uses categorised as complex, i.e., they could neither be identified as non-essential nor essential uses, accounted for 35% of the total uses. They summed up to approximately 7,685 tonnes per year, accounting for 78% of the total use volume (Figure 2). These uses were categorised as complex because the information provided in the applications as well as the framing of the criteria posed a challenge in categorising the uses. Both types of challenges often occurred simultaneously. Representative examples of complex uses of each type are described in the following.

#### 3.4.1 Information provided in the applications: multiple purposes of the technical function and too broad information provided for end-uses

One reason for assigning a use to the complex category was due to the different purposes of the technical function in the context of the end-uses that were both essential and non-essential. Another reason was that too broad information was provided in applications for authorisation regarding the end-uses related to the SVHC. Both reasons led to the categorisation of uses of chromium trioxide as complex, which accounted for the majority of complex cases (74.3%, 26/35). The substance was mainly used as an etching agent of plastic or as a corrosion inhibitor on plastic and metal articles, also referred to as functional chrome-plating with decorative character (16/26, e.g., applications 0032–03, 0095–03, 0212–01, 0212–02, 0237–01). It should be noted that a couple of applications used several terms (e.g., “*Hardener/oxidising agent/colouring agent*” in application 0213–01), or a phrase (“*Ingredient used in functional chrome plating with decorative character processes to deposit metallic chromium,*” e.g., applications 0095–03, 0130–01, 0131–01, 0132–01 and 0133–01) to describe the same technical function. Most applications covered sanitary equipment, but also non-sanitary articles used in the automotive (brand labels, gear lever knobs, trim strips, etc.), cosmetic (perfume caps, scissors, etc.), household (shavers, parts of coffee machines, etc.) and furniture sector (chairs, kitchen furniture, etc.), as well as white goods such as frames of washing machine doors, interior parts in fridges, and such for general engineering like electrotechnical parts, microscopes, laser optics, etc. (e.g., 0032–03, 0064–02, 0095–03, 0210–01 and 0210–03). The sanitary equipment comprised articles such as bathroom taps, shower heads, towel rails, soap dishes, and mirror frames made for consumers, public facilities and professionals. In these applications, the purpose of the technical function was to ensure drinking water safety, as sanitary articles are deemed to be in contact with drinking water, by preventing nickel leakage and providing corrosion and wear resistance. Hence, hygiene can be maintained, and the sanitary equipment complies with regulations on drinking water quality, thereby fulfilling the criterion *Prevention […] of severe health issues: Unsafe water* (Table 1). However, it was indicated that the other purpose of the technical function, as a corrosion inhibitor, was needed to produce an aesthetic and visually appealing surface according to customer demands in a broad spectrum of sanitary and non-sanitary equipment which does not fulfil the essential use criteria. First, the latter purpose (aesthetics) of the technical function does not fulfil the essential use criteria compared to its former purpose (drinking water safety), and second, the purposes of the technical function and their relation to the essential use criteria could not be assessed due to a too broad spectrum of articles (i.e., end-uses) for which the SVHC was used. In application 0032–03, the applicants noted that “*not all manufacturers and/or importers and formulators were able to identify the end-uses of their raw materials or as job platers might do chrome plating for different end-uses*” (ECHA, 2016). Further, the applicant commented on the draft RAC and SEAC opinion that “*[i]n upstream applications there is increased potential for uncertainty. The uncertainty is* “*systemic.*” *SEAC itself acknowledges the problems of uncertainty such as broad uses across several industry sectors and inevitable variations in operating conditions between facilities in the draft opinion*” (ECHA, 2016). SEAC responded that “*SEAC agrees that uncertainties cannot be avoided in applications for authorisations. SEAC acknowledged this in its draft opinions but, additionally, highlighted the fact that some of the uncertainties present within this application are not due to the nature of applications for authorisations themselves, but rather to the approach chosen by the applicant (e.g., the broad scope, the approach for assessing economic impacts, etc.). The committees informed the applicant about these uncertainties already during the opinion-development stage*” and provided the webpage link to the guidance on uncertainty analysis (ECHA, 2016).

Other examples of a too broad and unclear end-use spectrum are the applications 0211–01, 0221–01 and 0221–02. Here, chromium trioxide or sodium dichromate was used to chrome-plate electrolytic tin plate (ETP), also referred to as tin-plated steel, used to produce food contact material, such as cans. As a corrosion inhibitor, chromium trioxide stabilises the surface of tin-plated steel sheets by forming a stable, inert oxide layer of metallic chromium and chromium (III) oxide to ensure corrosion resistance, and thus to protect food against light, air and contaminants during storage. In case of an absence of the technical function, potential negative impacts on consumers in terms of duration of the shelf-lives and lower aesthetic quality of the food. However, not all their tin-plated steel sheets are used in the food sector. The applicants indicated that 50% (“*primarily*”) of the tin-plated sheets “*are used among customers in the food packaging and processing sectors*” while non-food contact materials, such as aerosol sprays and cans for paints, accounted for less than 5% of the tin-plated steel applications (ECHA, 2021). Thus, even though the technical function provides corrosion inhibition and stability to the tin-plated steel sheets to food contact materials, it was impossible to identify if the technical function was essential among the spectrum of applications of these metal sheets.

#### 3.4.2 Framing of the essential use criteria: implicit wording and a list of general examples lead to difficulties in unambiguously interpreting the essential use criteria

Another reason for categorising uses as “complex” was because of difficulties in unambiguously interpreting the essential use criteria. For example, in application 0234–02, chromium trioxide was used to chrome-plate printing and embossing cylinders, also called gravure cylinders, used in the rotogravure printing and embossing industry (functional chrome plating). The applicant supplies “*customer-specific complete plating systems for different printing segments, including packaging, decorative, publication and embossing.*” They further describe that “*[r]otogravure is used primarily for long printing runs in applications such as magazines, catalogues, inserts, flyers, gift-wrap, and labels,*” labels for bottles, blister packs and security printing (bank notes) as well as to produce “*packaging, wallpapers and floorings, among many others*” (Maschinenfabrik Kaspar Walter GmbH and Co, 2011). Used as a plating agent, chromium trioxide provides a scratchproof, highly wear and corrosion-resistant surface of the gravure cylinders to achieve “*fine and clear images and high printing consistency,*” making the cylinders “*resistant to oxidation, enabling the use of water as a printing ink solvent*” fulfilling customer demands (Maschinenfabrik Kaspar Walter GmbH and Co, 2011). Even though the function of chromium trioxide is needed to achieve the desired technical requirements, we failed to unambiguously interpret the essential use criteria as they did not cover a use scenario like this. For example, the purpose of the technical function for proper newspaper printing could fulfil the criterion *Provision of resources or services which are critical for society: Communication infrastructure* (Table 1) and the labelling of bottles could fulfil the criterion *Sustainment of basic conditions for human life and health: Food security* (Table 1). It was however unclear whether there was a dependency on the technical function and whether there would be substantial impacts on consumer health and the functioning of society. Further, like in other uses of chromium substances as corrosion inhibitors, the applicant adds that the corrosion resistance would benefit the sustainability and durability of the plated articles. It was left to interpretation if these two aspects could fulfil the criterion *Protecting and restoring the natural environment,* or if this was rather a side-effect of the technical function.

Application 0219–01 covered the use of chromium trioxide in functional chrome-plating of cutter links of chain saws. The technical function as a plating agent ensures enhancement of the stay-sharp time of the cutter links and an easy grindability of the saws used in forestry work. Reliable equipment in forestry work might be considered indispensable for society, potentially fulfilling the criterion *Provision of resources or services which are critical for society: Obtaining critical raw materials* (Table 1). However, this or any other criterion was not sufficiently explicit to decide if it was fulfilled by this use and its technical function. It was unclear whether an absence of the technical function would have any unacceptable negative impacts on the functioning of society, i.e., whether society depends on the technical function of chromium trioxide as a plating agent on chain saw cutter links.

## 4 Discussion

### 4.1 Challenges in assessing essentiality based on the information provided in applications for authorisation and existing essential use criteria

In the present analysis, the essential use criteria as suggested in the WSP report were applied. According to the assumptions made and our interpretation of the criteria, 10% of the selected uses would be categorised as non-essential. It suggests that it is possible to use the essential use criteria for identifying non-essential uses of SVHCs under the REACH authorisation procedure and that authorisation may not have been granted in these cases if the essential use criteria had been applied. Uses identified as essential accounted for 55% of the analysed applications indicating a potential coherence between the current criteria for authorising a use under REACH and the essential use criteria. It should be noted that the analysis reported in this paper is based on applications submitted under the current system. If the essential use criteria were to be formally applied in the authorisation process, it is possible that the applicants would further develop their arguments in favour of essentiality.

Approximately one-third of the analysed applications were identified as complex. The insufficient information provided in the applications was one reason for that; an indication of multiple purposes of the technical function and too broad information on end-uses made it challenging to identify clearly non-essential uses. This contrasts with an analysis by Figuière and colleagues (Garnett and Van Calster, 2021) who suggested that sufficient information to assess the essentiality of a use is made available due to the data requirements in REACH. Given that the complex uses comprised 80% of the total use volume investigated in the present analysis, a decision in favour or against essentiality can have clear consequences for the exposure of the respective SVHCs. The uses identified as complex show that 1) guidance is needed for applicants on how to assess the impacts of not continuing to use an SVHC on the health/safety and functioning of society, and 2) applicants need to provide additional data on the final products/articles to enable understanding of the specific purpose of the technical function of the SVHC. However, upstream applications with a broad spectrum of end-uses, can be challenging.

As already mentioned in the WSP report, the essential use concept could be integrated into the REACH authorisation procedure as a complementary element to an application for authorisation, requiring companies to explicitly describe why they consider their use(s) essential without making the decision dependent on it, given the (legal) uncertainties of this novel approach to risk management (WSP E&IS GmbH, 2023). This option could be a step in gaining first experiences and re-evaluating the potential benefits and challenges of that concept regarding the information provided by the applicants. It remains to be tested whether adequate guidance for the essentiality assessment could facilitate the assessments as the applicants already do crucial preparatory work within the socio-economic analysis.

The uses were categorised as complex due to a lack of clarity in the essential use criteria we tested, i.e., those related to the necessity for health/safety and the criticality for the functioning of society. Implicit wording and a list of generic examples made the uses difficult to interpret unambiguously. However, practicability and clarity of the essential use criteria are needed to reduce subjectivity and personal bias. More empirical evidence is needed to learn from practice, i.e., to assess whether the essential use concept adds value to the decision-making in terms of reducing the time taken or improving the protection of human health and the environment. WSP acknowledged that criteria defined too broadly could lead to ambiguity and hence a more uncertain and less predictable procedure to identify and phase out non-essential uses. Some of the uses identified as complex exemplify that the essential use criteria were not sufficiently explicit, and thus not practicable to make a clear decision. The challenge of developing unambiguous essential use criteria, i.e., how *use*, *health*, *safety*, *functioning of society*, *essential*, *necessary*, and *critical* are defined, and their interactions evaluated, probably originates from the fact that this approach to regulating chemicals is based on more social-related values that are difficult to quantify and measure. In the *Late Lessons from Early Warnings* reports (European Environmental Agency, 2002), (European Environmental Agency, 2001), the European Environment Agency advised risk regulators to use non-expert knowledge in their assessments and to consider different assumptions and values among social groups to identify potential conflicts that might require action on early warning signs. Therefore, to achieve regulatory success and broad societal support for the essential use concept, Suffill and colleagues (Suffill et al., 2024) urged the inclusion of social data in the development of this concept. Examples include the lack of well-defined criteria and clarity on how to deal with economic considerations or differences in judgements of essentiality between citizens, but also between experts and lay people. They suggested using tools from the social and behavioural sciences to generate and analyse quantitative and qualitative different social perspectives. For example, the Mental Models Approach to Risk Communication (MMARC) provides an approach to systematically explore “*how experts and non-experts think about a given issue […] which help to identify overlaps and differences between groups, and from which best practice communications (e.g., for the public) can be developed to foster understanding and engagement*” (Suffill et al., 2024).

Further, values are a concept of beliefs that is mainly used in disciplines like sociology, philosophy, psychology and anthropology to describe and explain social and personal organisation. Ten basic values have been recognised across cultures, such as the appreciation for self-direction and security, or benevolence for others (Schwartz, 2012). Extensive research has shown that these values are widely shared among different societies in different countries globally and within Europe (i.e., high within-country consensus, small in-between-country differences), suggesting that culture does not determine values (Fischer and Schwartz, 2011). Rather, the relatively consistent hierarchy of values across societies might be explained by their importance for “*a smooth functioning of significant groups or the larger society*” (Schwartz, 2012). Mason-Renton and colleagues recommended investigating more into how values in policy for risk management could be identified, transparently communicated and incorporated into procedures (Mason‐Renton et al., 2018). In addition to that, the Joint Research Centre (JRC) of the European Commission states that a political decision that is value-motivated rather than opinion- or belief-motivated, is more supported across citizens, and that transparent decision-making is key to creating and upholding institutional trust and support (Scharfbillig et al., 2021). Thus, understanding basic values and their relative importance to citizens can clarify the essential use criteria and reduce ambiguity when defining what technical function of a hazardous chemical in a specific use would be considered *necessary*, and/or *critical* to health, safety and the functioning of society. In doing so, it might become possible to better distinguish between *essentiality* and *benefit*/*nice-to-have*. Potentially, the terms *necessary*, *critical* and *essential* could be considered as synonyms, which would help to simplify the definitions and make the concept of essential use easier to understand. It might also be possible to create a scale of essentiality to represent reality in the best way possible. In connection to that, what contributes to the wellbeing of citizens and what value citizens attach to the degree of performance of goods and services changes over time and might need to be monitored to adjust the criteria. For example, in a very first survey of that kind, EU citizens were asked about their opinions on the (non-)essentiality of uses of persistent chemicals. Uses related to personal care, household products and recreation were mostly rated non-essential, while uses related to building/construction and safety were mostly rated as essential (Karinen et al., 2024). Potentially, basic values theory and the inclusion of social data and the perspectives of non-experts could further help to explore citizens’ values and guide the political decision-making process on complex use cases and (non-)essentiality to find consensus in modern society on how to define and assess essentiality. Regardless of how we reach a common understanding, with the help of experts, citizens, or a combination of these, the role of bias and conflict of interests must be managed (Schäffer et al., 2023). Experts must be chosen carefully since they may have vested interests. The views of citizens must also be interpreted carefully since they too can be affected by vested interests via advertisements, social media campaigns and other channels (Suffill et al., 2024).

Social data could benefit the development of guidance on how to weigh the economic impacts of restricting non-essential uses. Without further specification, the European Commission stated that it does not intend to change current procedures of assessing economic feasibility, e.g., the socio-economic analysis under REACH that weighs the benefits against the risks of using a harmful chemical and its potential alternatives (ECHA, 2011b). Ideally, economic growth and the wellbeing of citizens and the environment should not be mutually exclusive. However, the prioritisation of for-profit activities has been criticized for becoming “commercial determinants of health” (Lee and Freudenberg, 2022). The widespread use of PFAS exemplifies how a class of chemicals with impressive technical properties led to economic growth and societal wealth, but at the same time posed known and unknown health risks in the long-term. Now it is known that there are considerable societal costs associated with PFAS for healthcare, clean up, litigation, etc., and that these costs are predicted to far exceed corporate financial benefits (Goldenman et al., 2019), (Cousins et al., 2022). Therefore, in future decision-making, (short-term) corporate and national financial interests needs to be weighed against potential long-term societal costs associated with chemical pollution.

### 4.2 The current ECHA use descriptor guidance should be revised to enhance its usefulness for a coherent, transparent and protective essentiality assessment

Aligning the terminology for the technical function would add consistency and comparability of uses to the decision-making process. Often, different terms or phrases were used to describe a technical function although the purpose that the SVHC fulfilled was the same. For example, in the application 0190–01 both terms “*surfactant*” and “*emulsifier*” were used. It needs to be clarified if these technical functions differ from one another or if that is a case of inconsistent wording. In 2017, the Organisation for Economic Co-operation and Development (OECD) published a proposal on *Internationally harmonised functional, product and article use categories* to facilitate and harmonise the descriptions of uses which is pivotal to assessing the exposure of humans and the environment to harmful chemicals (OECD, 2017). An essentiality assessment as well as the use descriptor guidance provided by ECHA could benefit from a refined description of the technical function in the different stages of a use based on the OECD guidance.

In 2015, Tickner et al. (2015) suggested the approach of functional substitution to identify alternatives to harmful chemicals that provide the same functions as the target substance. They introduce a more sophisticated scheme to define the function of a chemical substance which goes beyond its technical function. The authors encourage considering three different levels of functions when trying to identify potential alternatives to a chemical of concern: (1) the chemical function, which is defined by the physicochemical properties of the substance of interest (i.e., the technical function); (2) the end-use function which describes the purpose served by the chemical function in a specific product or process; and (3) the function as a service which describes the service provided the chemical function in the specific application to society. Using this scheme, it could be evaluated if the technical performance of a certain product or process depends on the technical function provided by the substance of interest. In the present analysis, the descriptions of the product, article and environmental release categories according to ECHA’s use descriptor system (ECHA, 2015) did not always provide the descriptions needed to fully describe the purposes of SVHCs across its entire life cycle for identifying non-essential uses. For example, the raw chemical chromium trioxide contains hexavalent chromium which is responsible for the carcinogenic effect of the substance. It is part of a chemical product that reacts with the surface of articles during the plating process. As soon as chromium trioxide reacts, it is reduced to elementary chromium which is not toxic *per se*. Thus, workers are at risk but not consumers as the final article does not contain the substance of concern. But to identify non-essential uses of hexavalent chromium trioxide, the final articles (e.g., sanitary equipment) that result from using the substance and that contain elementary chromium need to be considered. Thus, ECHA’s use descriptor system could benefit from implementing a descriptor system of the functions of the substance as suggested by Tickner and colleagues to better describe the purposes fulfilled by an SVHC along its life cycle.

Further, despite considering the context of the end-use, e.g., a final article, the current analysis employed the essential use concept to applications for authorisation in a potentially narrow way, i.e., only the end-use function instead of the function as a service was considered, as described by Tickner et al. (2015). However, assessing uses only at the end-use level, may not lead to complete phase-out of that SVHC. For a reasonable protection of workers, consumers and the environment as well as possibly enhancing circularity and reducing waste, the function of an SVHC would need to be considered as a service, to assess if a system change is possible (Tickner et al., 2015). For example, even if the technical function of an SVHC in a certain article may be essential, there might be alternative articles that provide the same service as the SVHC without depending on the SVHC. This could be the case in applications 0211–01, 0221–01 and 0221–02 in which chromium trioxide or sodium dichromate were used to chrome-plate tin plates partially for the production of food contact material, such as cans. The purpose of the technical function was to ensure food safety. When considering the technical function as a service, it should be investigated if other food packaging materials could provide the same service without depending on the SVHC.

## 5 Summary and recommendations

In the present analysis, a pool of 100 REACH applications for authorisation was retrospectively analysed based on the essential use criteria on the necessity for health, safety and the criticality for the functioning of society. Following assumptions made and our interpretation of the criteria, 10% of the uses were identified as non-essential. Examples include chromium VI substances used to plate plastic and metal articles to provide a visually appealing surface for the final article. Essential uses accounted for approximately 55% of the uses. These were mainly uses of the nonylphenols NPnEO and OPnEO as surfactants or emulsifiers in the manufacture of pharmaceutical and medical products. Approximately one-third (35%) of the analysed uses were categorised as complex, meaning that no clear categorisation as essential or non-essential could be made. This was partly due to the information provided in the applications for authorisation. It was challenging to clearly identify non-essential uses due to the multiple purposes stated for the technical function and the broad end-use information. The categorisation of uses as complex also stemmed from the definition of the essential use criteria. Here, implicit wording and a list of general examples made it difficult to clearly interpret the essential use criteria.

Practicability and clarity of the essential use criteria are needed to reduce subjectivity and personal bias. To validate and continuously improve the criteria, we recommend integrating experience from practice, e.g., by running iterative trials in which respective stakeholders give feedback on how practically and clearly the criteria were perceived. Further, ECHA’s use descriptor system was of limited use in describing the uses in the context of an essentiality assessment as it does not include a description of the relevant stages of a use and its technical function, e.g., the technical function required for industrial use *versus* the technical function for consumer use. We therefore recommend that ECHA reviews its current use descriptor system based on the current OECD guidance. In addition, we recommend developing clear guidance on how to weigh short-term economic impacts of restricting non-essential uses against long-term societal costs. Further refinement of the essential use criteria by incorporating social data, such as, e.g., consumer perspectives, and basic value theory to enhance the practicability and societal support of the criteria while reducing ambiguity, is also needed. To ensure that applicants for authorisation transparently and systematically provide the information needed for an essentiality assessment technical guidance for applicants is needed.

## Data Availability

The original contributions presented in the study are included in the article/Supplementary Material, further inquiries can be directed to the corresponding author.
