# Supplementary material for: Identifying non-essential uses to phase out substances of very high concern under REACH
Source: Front Toxicol. 2024 Nov 1;6:1488336. doi: 10.3389/ftox.2024.1488336 (PMC11564159; doi:10.3389/ftox.2024.1488336)
Supplement: Supplementary file 1 [file Table2.docx]

Supplementary Information to

**Identifying non-essential uses to phase out Substances of Very High Concern under REACH**

Flora Borchert^1*^, Romain Figuière^1^, Ian T. Cousins^1^, Christina Rudén^1^, Marlene Ågerstrand^1^

^1^ Department of Environmental Science, Stockholm University, Stockholm, Sweden

*Corresponding author: [flora.borchert@googlemail.com](mailto:flora.borchert@googlemail.com)

**2. Method**

***2.1 Scope, assumptions and definitions of the essential use concept in the present analysis***

The stepwise horizontal approach to an essentiality assessment of a use as suggested by the European Commission and refined by WSP in their report was employed in the present study as the following:

1. *Scoping: Is the essential use applicable?*

It should to be determined whether first, the substance is “*one of the* *“most harmful chemicals” for which phasing out is a priority as per the CSS* [the Chemical Strategy, note by the authors]”, and second, whether the substance is “*used in an article, mixture, product, process, or service*”. The REACH Authorisation procedure currently addresses SVHCs which are a subset of *the most harmful chemicals* as defined by the European Commission [1]. Thus, the present analysis focused on substances listed as SVHCs in REACH Annex XIV. The essential use concept is further understood as to address the *use* of a substance, thus, not the substance, product or article *per se* [2]. According to REACH article 3.24, “use” is defined as “*any processing, formulation, consumption, storage, keeping, treatment, filling into containers, transfer from one container to another, mixing, production of an article or any other utilisation”* [3]. Further, under REACH, a use is defined as an end-use when “*the substance: has reacted (therefore it does not exist anymore in its original form)*, *or has become part of an article, or has completely been released via waste water or exhaust air, and/or it is contained in waste from this use*” [4]. However, while the REACH Regulation defines *use* in more practical terms, the essential use concept rather addresses the technical function provided by a substance and, in a societal context, if it is needed for a specific end-use which can be a final product or article used by professionals or consumers during service life [2]. A substance’s technical function is the specific task it fulfils in the end-use, i.e. to provide specific properties to the final product or article.

1. *Assessing essentiality*

The assessment of the essentiality addresses two steps. Step 1: Assessment of the necessity of a use for health/safety and the criticality of a use for the functioning of society, and Step 2: Assessment of alternatives to identify the ones that are acceptable from the standpoint of environment and health. In the WSP report, it is emphasized that “*Contextualising the use in terms of its necessity for health or safety/criticality for the functioning of society is imperative*”, so the use “*should be assessed through considering the societal need for the technical function provided by the most harmful chemical in a specific end use (e.g. final product) in a defined setting*” ([2], page 5 and 7, as well as 38/39). Consequently, our analysis focussed on the technical function of an SVHC that fulfils a certain purpose in a particular end-use e.g. being a component in a chemical product (formulation/mixture), in/on an article, or process to manufacture certain products, hence, covering any uses “*where the substances are incorporated in the product, as well as uses of substances to produce the final product but where the substances do not remain in the product itself*” [2]. “Substance function” and “chemical function” were considered synonyms for technical function [2], [4]. However, no assessment of step 2, i.e. the assessment of acceptable alternatives, was performed. Indeed, an inherent part of the current REACH Authorisation procedure is the assessment of alternatives and the provision of a substitution plan. We assumed that there are no ready-to-use alternatives available to the applicant that provide the same or a comparable technical function and/or a lower toxicity and/or risk by the time they apply for authorisation. How to improve alternatives assessment overall and how *acceptable* alternatives under the essential use concept would be defined is the subject of ongoing research and discussions [2], [5], [6]. Consequently, we framed the scope of the present analysis on the health/safety and functioning of society aspects of the essential use concept only. Further, the sufficiency and reliability of the data analysed were not addressed and would need to be addressed in future research.

Economic impacts on the company or the wider economy were not included in the analysis as they are currently not explicitly mentioned in the criteria according to the latest information published in the WSP report [2]. The socio-economic analysis for authorisation under REACH includes economic impacts on the applicant (manufacturer, supplier, downstream user) which, however, does not match the narrative of the essential use concept ([7], see also Supplementary Information Table S2). If and how this should be changed is not the scope of the present analysis.

1. *Decision-making and setting conditions for use*

This step foresees that essential uses of a substance are authorised with specified conditions for a specific time, while non-essential uses of that substance are not allowed (not authorised). Our analysis focused on the identification of non-essential uses which are deemed to be prioritised for phasing-out. Therefore, we aimed at categorising the uses as non-essential or essential based on the information provided in applications for authorisation. We also categorised cases where the decision-making, based on the current criteria and the information provided, was not as straightforward as *complex* (see section 2.4.2). However, we did not suggest conditions for use.

1. *Review*

Before an authorisation expires, the (non-)essentiality of a use may be reviewed when there is progress concerning the availability of alternatives and/or the societal need for a use (technical function) changes. Otherwise, the granted authorisation expires and the use is banned. In the present analysis, we did not discriminate between initial applications and review reports where the applicant applied for a prolongation of the earlier granted authorisation. How decisions change over time and how an essentiality assessment contributes to prolongations or expirations of uses could be topics of future research.

**Table S2**. Definition of the adverbs *necessary* and *critical*, according to the WSP report.

| Adverb | Definition |
| --- | --- |
| Necessary | “*meaning that uses which are only somewhat related to the health/safety criterion, e.g. uses which provide a low level of benefit or convenience, should not be deemed necessary for health/safety.* ***Only uses upon which health and safety are dependent should be considered as “necessary*** ***for health and/or safety”.*** *Consideration of the severity of potential impacts of withdrawal of uses on health and safety* ***(but not on other socio-economic considerations)*** *may help to clarify this assessment but not in isolation*.” |
| Critical | “*meaning that* ***only uses upon which the functioning of society is dependent on*** ***should be deemed critical for the functioning of society.*** *Uses which are only somewhat related to the criterion e.g. uses which provide a low level of benefit or convenience, should not be deemed critical for the functioning of society. Consideration of the severity of potential impacts of withdrawal of uses on the functioning of society* ***(but not on other socio-economic considerations)*** *may help to clarify this assessment, but not in isolation*.” |

- 1. ***Analysis of the selected applications for authorisation***

Note: If information was not clear or missing, the Chemical Safety Report, in particular, sections 9 (exposure assessment) and 10 (risk characterisation), were scrutinised as well. Please note the SEA documents were the non-confidential summaries provided by the applicant on ECHA’s webpage.

***Information collected from the applications for authorisation***

The following general information was collected from the selected applications for authorisation (section 2.3):

- *Type of actor and country of origin*: The applicant can be a manufacturer, importer, formulator or downstream user. Name of the country where the applicant is located.
- *Supply chain coverage*: The applicant can either apply for the own use(s) and/or the downstream use(s).
- *Tonnage band*: A number in tonnes per year that the applicant seeks to continue using.
- *Application for authorisation route*: The applicant can either demonstrate that the risks are controlled (adequate control route), or that the socio-economic benefits outweigh the risks to human health and the environment (socio-economic route).
- *Review period recommended by the applicant and by RAC and SEAC*: Number of years for how long an authorisation is recommended to be granted by the applicant and RAC/SEAC, respectively, and after which a review of the existing authorisation should be performed in case the applicants seek to prolong the authorisation.

The following information on the end-use was collected:

- *Sector of end-use* as described by the applicant according to the ECHA use descriptor system [4].
- *Use name* as described by the applicant.
- *Chemicals Product category* of the end-use according to the ECHA use descriptor system as indicated by the applicant, e.g. laboratory chemical, detergents, paints or metal surface treatment product. The chemical product “*describes in which types of chemical products (= substances as such or in mixtures) the substance is finally contained when it is supplied to, and used by, end-users*” [4]. If no product category was provided by the applicant, the most appropriate description according to the available information was indicated.
- *Article category* of the end-use according to the ECHA use descriptor system as indicated by the applicant and related to the subsequent service life of the end-use, e.g. metal articles or textiles. The article category describes “*the types of article in which the substance is contained or on which the substance has been applied*”, including “*mixtures in their dried or cured form (e.g. dried printing ink in newspapers; dried coatings on various surfaces)*” [4], [7]. If no article category was provided by the applicant, the most appropriate description according to the available information was indicated. While the product category describes the consumer activities and/or market information for workers’ uses, the article category describes the service life activities and uses for consumers and/or workers [4].
- *The user of the final end-product* during its service life that results from using the SVHC. This could be industrial, professional and/or consumer end-users.

The following information on the technical function was collected:

- *Technical function* (TF) provided by the SVHC as indicated by the applicant. Technical function is synonymously used for substance function and describes the role that the SVHC fulfils in a specific use [4].
- *Critical properties of the SVHC*, i.e. key functionalities like technical requirements, performance and/or physio-chemical properties the substance must provide to ensure that the final product or article is given a specific performance and properties, as indicated by the applicant.
- Indication of whether *legal requirements* (e.g. regulations) and/or *customer requirements* (e.g. certain product qualities and specifications) require the technical performance of the SVHC related to its function in/for the final product or article.
- *Type of technical performance assessment* of the chemical product or article in/for which the SVHC was used. This can be qualitative and/or quantitative specifications.

**References**

[1] European Commission, “COMMUNICATION FROM THE COMMISSION TO THE EUROPEAN PARLIAMENT, THE COUNCIL, THE EUROPEAN ECONOMIC AND SOCIAL COMMITTEE AND THE COMMITTEE OF THE REGIONS Chemicals Strategy for Sustainability Towards a Toxic-Free Environment,” 2020.

[2] WSP E&IS GmbH, “Supporting the Commission in developing an essential use concept - Final Report,” 2023.

[3] European Commission, “Regulation on the Registration, Evaluation, Authorisation and Restriction of Chemicals (REACH) EC Nr. 1907/2006,” no. December, 2006.

[4] European Chemicals Agency, *Guidance on information requirements and chemical safety assessment Chapter R.12 : use description, version 3.0 - December 2015.* ECHA, 2015.

[5] J. A. Tickner, J. N. Schifano, A. Blake, C. Rudisill, and M. J. Mulvihill, “Advancing safer alternatives through functional substitution,” *Environ. Sci. Technol.*, vol. 49, no. 2, pp. 742–749, Jan. 2015.

[6] ECHA, *Impacts of REACH Restriction and Authorisation processes on substitution in the European Union*. 2020.

[7] ECHA, “Guidance on the preparation of socio-economic analysis as part of an application for authorisation,” 2011.
